# Running Speed and Mental Toughness: Effects on Change-of-Direction Speed in Police Students

**DOI:** 10.3390/jfmk11030268

**Published:** 2026-07-08

**Authors:** Ranko Rajović, Nenad Koropanovski, Filip Kukić, Igor Radošević, Miloš Milošević, Milivoj Dopsaj

**Affiliations:** 1 Department of Neuroscience in Education, Faculty of Education, University of Primorska, 6000 Koper, Slovenia; ranko.ntc@gmail.com; 2 Department of Criminalistics, University of Criminal Investigation and Police Studies, 11000 Belgrade, Serbia; korpan82@gmail.com; 3 Faculty of Physical Education and Sports, University of Banja Luka, 78000 Banja Luka, Bosnia and Herzegovina; filip.kukic@gmail.com; 4 Faculty of Physical Education and Sports Management, Singidunum University, 11000 Belgrade, Serbia; iradosevic@singidunum.ac.rs; 5 Faculty of Sport and Physical Education, University in Belgrade, 11000 Belgrade, Serbia; milivoj.dopsaj@gmail.com

**Keywords:** psychological resilience, neuromuscular fatigue, tactical readiness, agility performance, psychophysiological stress, law enforcement recruits

## Abstract

**Objectives**: Effective operational functioning within the tactical domain requires a high integration of physical and psychological capacities under stressful conditions. This study investigated the impact of physical exertion at varying intensities (anaerobic and aerobic running) on subsequent change-of-direction speed (CODS) performance among police students, while evaluating the role of mental toughness (MT) and biological sex. **Methods**: Thirty police students (36.7% female) completed running protocols at different intensities (300-yard shuttle run and 2.4 km Cooper test), immediately followed by the Illinois Agility Test (IAT) to assess CODS performance. Mental toughness was evaluated using the Mental Toughness Index (MTI). Repeated-measures ANOVA and ANCOVA were used to analyze the main and interaction effects. **Results**: Initial repeated-measures ANOVA revealed that increasing running intensities significantly degraded CODS performance, demonstrating large main effects for both the anaerobic (F = 56.70, *p* < 0.001, η^2^_p_ = 0.661) and aerobic (F = 47.50, *p* < 0.001, η^2^_p_ = 0.621) protocols. After introducing mean-centered MT as a covariate, the main effect of Running Speed remained highly significant in both the anaerobic (F = 57.52, *p* < 0.001, η^2^_p_ = 0.673) and aerobic (F = 46.01, *p* < 0.001, η^2^_p_ = 0.622) models, with no significant univariate interaction effects involving MT or sex on the rate of decline. Mixed ANCOVA showed a significant main effect of sex on absolute IAT times under both anaerobic and aerobic conditions, with males consistently outperforming females. No significant differences in baseline MT scores were observed between sexes (*p* = 0.507). **Conclusions**: The findings demonstrate that physical fatigue robustly impairs CODS performance at a parallel rate for both male and female police students. Mental toughness does not neutralize the physiological rate of performance decline under acute fatigue. Practical training programs should integrate physical and psychological conditioning scenarios, while workload management protocols can be uniformly applied across biological sexes.

## 1. Introduction

Change-of-direction speed (CODS), defined as the ability to rapidly change direction of movement in a preplanned manner, is a critical component of physical performance in tactical occupations such as law enforcement [1]. Police officers are frequently required to perform explosive multidirectional movements during operational tasks, including suspect pursuits, obstacle negotiation, and rapid tactical repositioning [2,3]. Importantly, these movements are rarely executed under rested conditions, as they are commonly preceded by high-intensity anaerobic or prolonged aerobic exertion [4]. Previous studies have demonstrated that accumulated neuromuscular and metabolic fatigue substantially impairs subsequent CODS performance, reducing movement efficiency and operational readiness under physically demanding conditions [4]. In addition, sex-related physiological and anthropometric differences may further influence CODS performance and fatigue responses during tactical tasks, with male officers generally demonstrating superior acceleration, deceleration, and load carriage capacities compared to females [3,5]. Consequently, understanding the factors that may attenuate fatigue-induced declines in CODS performance is of considerable importance for optimizing tactical preparedness and occupational safety.

Although physiological conditioning plays a central role in maintaining operational performance under fatigue, growing evidence suggests that psychological factors may also substantially influence physical task execution during acute physiological stress. Among these factors, mental toughness (MT) has emerged as an important psychological construct associated with enhanced resilience, persistence, and the maintenance of performance under demanding conditions [6,7]. Traditionally conceptualized through the 4Cs framework as a relatively stable characteristic involving control, commitment, challenge, and confidence [8], MT is increasingly described in contemporary perspectives as a dynamic psychological resource that may influence how individuals perceive, tolerate, and respond to physical fatigue [9]. Previous research has shown that individuals with higher MT often demonstrate superior tolerance to exertion, lower perceived fatigue, and greater capacity to sustain performance during physically demanding tasks [10,11]. Within tactical populations, these characteristics may be particularly relevant, as police officers are routinely required to preserve movement efficiency and task execution despite substantial physiological and cognitive strain. However, despite growing interest in MT within sport and performance psychology, limited research has examined its potential role in preserving CODS performance following acute aerobic and anaerobic exertion among police populations. Moreover, little is known about whether the influence of mental toughness on fatigue-related performance decline differs between male and female police students. Traditionally, mental toughness has been examined in relation to endurance performance, persistence, pain tolerance, and perceived exertion; its influence may extend to the maintenance of complex motor performance during acute fatigue. Successful execution of CODS under fatigued conditions depends not only on the preservation of force-generating capacity but also on effective central regulation of movement, including attention allocation, executive control, motor planning, and rapid neuromuscular coordination. Acute neuromuscular fatigue disrupts both central and peripheral mechanisms, impairing movement precision and coordination required for rapid directional changes [12]. Contemporary psychobiological models additionally suggest that performance under fatigue is influenced by cognitive regulation of effort and the ability to maintain goal-directed behavior despite increasing physiological and psychological demands [13,14]. Individuals with higher mental toughness consistently manifest superior attentional control, emotional regulation, resilience to stress, and the ability to maintain performance under demanding conditions [7,9,15]. Altogether, these findings provide a plausible neurocognitive framework suggesting that mentally tougher individuals may better preserve CODS performance following acute neuromuscular fatigue, even though explicit empirical evidence examining this relationship remains limited.

In light of the aforementioned arguments, the primary aim of this study was to explore the possible contribution of mental toughness to CODS performance under fatigue caused by maximal and submaximal anaerobic and aerobic running among police students. A secondary aim was to examine potential sex-related differences in CODS performance and mental toughness under these conditions. It was hypothesized that: (i) increasing running speed would significantly impair CODS performance; (ii) higher levels of mental toughness would attenuate fatigue-related declines in CODS performance at all running speeds; and (iii) the protective effect of mental toughness against fatigue-related performance decline would be more pronounced in male than in female police students. While the first hypothesis is directly derived from empirical precedents, the second and third are primarily driven by theoretical extensions due to the lack of direct evidence on whether cognitive framing and psychological resilience can buffer performance declines during brief, maximal explosive tasks. The findings of this study may inform the development of integrated physical and psychological conditioning strategies to improve operational readiness and fatigue tolerance in tactical populations.

## 2. Materials and Methods

### 2.1. The Participants

Our sample consisted of 30 students (36.7% female). The main characteristics for males were age = 19.7 ± 0.5 years, body height = 182 ± 4.9 cm, body weight = 80.2 ± 9.9 kg, and body mass index = 24.3 ± 2.4 kg/m^2^. The main characteristics for female participants were age = 19.7 ± 0.6 years, body height = 171 ± 5.7 cm, body weight = 62.4 ± 5.1 kg, and body mass index = 21.5 ± 2.0 kg/m^2^. All participants met the physical fitness requirements for recruitment and attended physical education classes (self-defense, use of force, and strength and conditioning) three times per week, which is why they are already familiar with the protocols we used in testing. They were informed about the aim of the study, testing protocol, and potential risks. Inclusion criteria were that the participant was in good health and willing to participate in the study. The exclusion criterion was the participant’s failure to perform all tests. Only participants who signed informed consent were included in the study. This study received ethical approval (440-2).

### 2.2. Study Design and Procedures

Data were collected across 10 testing sessions. During the first two testing sessions (Testing Days I and II), anthropometric and psychological data were collected, and participants were familiarized with the testing protocol. In addition, before each testing session, participants performed a 10-min warm-up consisting of running variations and calisthenics exercises. Testing sessions consisted of several testing stations that participants completed consecutively in the following order:

- DAY III: (1) Illinois Agility Test, (2) 5 min rest, (3) 300-yard shuttle run test;
- DAY IV: (1) Illinois Agility Test, (2) 5 min rest, (3) 2.4 km Cooper test (maximal effort), (4) Illinois Agility Test (no rest between 3 and 4);
- DAY V: (1) Illinois Agility Test, (2) 5 min rest, (3) 2.4 km Cooper test at 95% of maximal aerobic speed, (4) Illinois Agility Test (no rest between 3 and 4);
- DAY VI: (1) Illinois Agility Test, (2) 5 min rest, (3) 300-yard shuttle run with 95% intensity, (4) Illinois Agility Test (no rest between 3 and 4);
- DAYS VII, VIII, and IX: as per DAY VI above, but with the 300-yard shuttle run performed at 90, 85, and 80% of maximal effort, (4) Illinois Agility Test (no rest between 3 and 4).

For safety reasons, participants did not perform the IAT after the maximal SR300y because they were fatigued. The IAT was also repeated on DAY X for reliability purposes and to control for learning and training effects. Although incorporating a wider range of aerobic intensities, mirroring the multi-level anaerobic protocol, would have provided a more balanced experimental design and facilitated more comprehensive comparisons, this approach was strictly constrained by logistical limitations, restricted testing timelines, and the ethical necessity to mitigate excessive cumulative fatigue among police students, who already face intense daily physical demands during their institutional training. The order of submaximal intensities was randomized.

### 2.3. Settings

**Figure 1:**
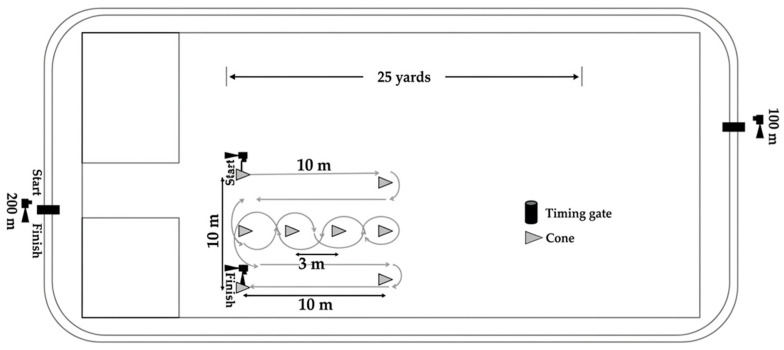
Schematic of test positioning.

This study was conducted at the University of Criminal Investigation and Police Studies, Belgrade, Serbia. Participants were accommodated on the university campus, which allowed for control of their exercise activities during the testing sessions and the rest periods between sessions. During the testing sessions, they had no other training activities. Therefore, they were sufficiently rested and had the same conditions. More specifically, the rest period between sessions was 3 days (72 h). The tests were conducted at the same time of day, in the same footwear and apparel, and under identical environmental circumstances (temperature 21 ± 2.1 °C, relative humidity 58%, barometric pressure 1001 ± 9 mb). Anaerobic endurance and CODS were assessed in a nonslip sports hall, while aerobic endurance was assessed on an outdoor circular track measuring 200 m (Figure 1). The distances between the finish lines of the anaerobic endurance test and the aerobic endurance test and the starting line of CODS were 5 m and 10 m, respectively. After the anaerobic and aerobic tests, participants walked swiftly to the CODS test starting line.

### 2.4. Measurement Procedures

#### 2.4.1. Morphological Characteristics

Body height (BH) was measured with a portable stadiometer (Swiss Instruments, Zurich, Switzerland), and body mass (BM) was measured using the InBody 720 device in accordance with a standardized procedure [16]. From the obtained values, BMI was calculated.

#### 2.4.2. Anaerobic Running

Maximal and submaximal anaerobic running were assessed using the 300-yard shuttle run test (SR300y). The test includes 12 lengths of 25 yards. It has previously been used with sports and tactical populations [17,18]. The test–retest reliability of this test (ICC = 0.83) has been determined elsewhere [19]. Individual times obtained from a maximal SR300y (SR300ymax) were used to calculate intensities of 95, 90, 85, and 80% (SR300y95, SR300y90, SR300y85, and SR300y80, respectively) for each participant in order to cover the range of anaerobic intensities (i.e., BLa > 4.0 mmol/L) [20]. For example, the equation for calculating the time for an 95% percentage of maximal speed was

Time for SR300y95 = time for SR300ymax + (time for SR300ymax × 0.05)(1)

The running speeds were governed by a visual light pacer (Pacer2, KulzerTEC, Santa Maria da Feira, Portugal), which consisted of a 22.86 m (25 y) long line equipped with 26 LED lights per meter, spaced from 0 to 22.86 m.

To enforce strict uniform pacing during the submaximal SR300y protocols, a custom-programmed, 25-yard addressable LED pacing strip was positioned flat on the floor parallel to the running lane. The system was programmed to flash sequentially down the 25-yard track and immediately reverse its direction upon reaching each turn line for a total of 12 lengths. Crucially, to accommodate the biomechanical demands of the 180° turns, the pacing software applied a non-linear velocity profile: the sequential flashing decelerated slightly within the final 3 yards approaching each turning boundary to allow for safe deceleration and turning mechanics, followed by an accelerated phase over the initial 3 yards of the subsequent length to aid re-acceleration. Participants were instructed to maintain their torso in line with the moving wave of light throughout the test.

Each participant’s heart rate (HR) was monitored using a Polar V800 device (Polar Electro, OY; Kempele, Finland) secured with a chest strap. Maximal HR (Hrmax) was recorded at the end of each test to control for running speed. Blood lactate (BLa) levels were measured 3 min after the test, when BLa accumulation is reported to be at its highest [21]. BLa was measured only after the SR300ymax to ensure that participants provided maximal effort, as all other SR300y intensities were calculated based on SR300ymax. Capillary blood lactate was uniformly sampled exactly 3 min post-exercise for all participants, following established guidelines identifying this window as optimal for capturing peak lactate concentrations after maximal efforts. The mean HRmax was 188.98 ± 8.46 beats per minute while mean BLa 13.81 ± 1.66 mmol/L.

The primary mechanism used to guarantee that participants performed exactly at the prescribed submaximal intensities was strict external pacing control as well as measuring heart rate. Blood lactate was not sampled during the submaximal protocols due to logistical constraints.

#### 2.4.3. Aerobic Running

Maximal and submaximal aerobic running were assessed using the 2.4 km Cooper test (CT2.4km), which has previously been used in law enforcement recruits [22]. The reliability (ICC = 0.99) and accuracy (bias correction = 0.994 for distance and 0.956 for HR) of the Cooper running test were shown to be acceptable in long-distance runners [23]. The time required to complete the CT2.4km as quickly as possible (i.e., maximal effort) was used to calculate the 95% percentage of maximal aerobic speed. For example, to calculate 95% percentage of maximal aerobic speed, the following equation was used:

Time for CT2.4km95 = time for CT2.4km + (time for CT2.4km × 0.05) (2)

To operationalize this protocol for field monitoring, the final target time in seconds was divided by 24, since a 2.4 km run executed on a 200 m track comprises exactly 24 segments of 100 m. This calculation provided the exact split time in seconds required per 100 m. Two experienced instructors were positioned at 100 m intervals along the track and used these calculated split times to provide immediate verbal pacing feedback (e.g., to maintain pace, accelerate slightly, or slow down slightly), ensuring that participants strictly adhered to the designated submaximal speed. Introduced ranges were used to determine the effects of different aerobic intensities on subsequent CODS performance. As the participants were well familiar with the Cooper test, they were instructed to keep the pace as constant as possible during maximum performance. The same instruction was given when they ran at set intensities. Two experienced instructors were placed at 100 m intervals and provided clear feedback (e.g., keep up the pace, accelerate slightly, or slow down slightly) to ensure that participants ran at a set percentage of maximal speed. In addition, HRmax was measured immediately after the maximal CT2.4km to ensure that participants ran at maximal effort. The mean HRmax was 195.64 ± 9.95 beats per minute.

The primary mechanism used to guarantee that participants performed exactly at the prescribed submaximal intensities was strict external pacing control as well as measuring heart rate.

#### 2.4.4. Change-of-Direction Speed

Change-of-Direction Speed (CODS) was assessed using the standardized Illinois Agility Test (IAT) following procedures previously reported in the literature [18]. The time to complete the test was measured by timing gates (Fitro Light Gates, Fitronic, Bratislava, Slovakia) and expressed in seconds, with a precision of 0.01 s. The test was performed twice, and the better result was recorded for the analysis. The IAT was also repeated on DAY X for reliability purposes and to control for learning and training effects, given the number of testing sessions. To eliminate potential learning or practice effects during the multi-session experimental period, a mandatory familiarization was conducted prior to data collection, during which participants practiced the IAT layout until their execution times stabilized. The total timeframe from the first baseline testing to the final session spanned 4 weeks. Test–retest reliability was high (ICC = 0.985), which was similar to that (ICC = 0.96) reported in previous studies [24]. A paired-samples *t*-test confirmed that there was no statistically significant systematic difference between the initial baseline and subsequent unfatigued control assessments (t = 0.412, *p* = 0.681), demonstrating that learning effects did not confound the experimental outcomes across the sessions.

#### 2.4.5. Mental Toughness

MT was assessed by the Mental Toughness Index (MTI) [7]. The instrument consists of 8 items; each answered using a 7-point Likert-type scale. The total score ranges from 1 (False, 100% of the time) to 7 (True, 100% of the time). The construct validity of the MTI has been supported by studies involving participants from various cultures [25,26]. In previous studies, reliability estimates for MTI scores were α ≥ 0.86, which is in accordance with the results (Cronbach’s Alpha ≥ 0.81) of the validation study for assessment of police students [27]. The final MT score was calculated as the average value of participants’ answers to all items. This procedure has been shown to be valid in previous studies [28]. Since the MTI was administered only once at baseline, this study implicitly operationalizes mental toughness as a stable psychological trait rather than a dynamic, fluctuating state.

### 2.5. Variables

The effects of aerobic and anaerobic fatigue on CODS were assessed in absolute terms by comparing the initial IAT value (IATinitial) with the results of tests performed after aerobic and anaerobic running at different intensities (IATPost300y80, 85, 90, 95, and IATPostCT95, max). The effects were also assessed relatively, as differences between the pre- and post-running tests were calculated and expressed as percentages (EfIATPost300y80, 85, 90, 95, and EfIATPostCT95, max).

### 2.6. Statistical Analysis

An a priori power analysis conducted in G*Power (Version 3.2; Franz Faul) for repeated-measures ANOVA with within–between interaction effects (α = 0.05, power = 0.95, effect size f = 0.25, correlation among repeated measures = 0.85, ε = 1) indicated that a minimum sample size of 16 participants was required for a design with two groups and three repeated measurements, whereas 12 participants were required for a design with two groups and five repeated measurements. Given the planned inclusion of one covariate in the repeated-measures ANCOVA model, which increases the model’s degrees of freedom and consequently the required sample size, the minimum recommended sample size was adjusted to 17.

The data analyses were performed using JAMOVI (Version 1.2; The Jamovi Project) and SPSS (Version 23.0; IBM Corp., Armonk, NY, USA) statistical software. Descriptive statistics for both sexes were calculated, including the mean, standard deviation (SD), minimum (Min), and maximum (Max). The Shapiro–Wilk test was used to assess the normality of the distribution. To examine sex-related differences in MT, IAT performance, and relative fatigue-induced changes in IAT performance across all running conditions, independent-samples *t*-tests were conducted. To examine the effects of anaerobic and aerobic running intensities on subsequent CODS performance, separate repeated-measures analyses of variance (RM ANOVAs) were conducted, with running percentage of maximal speed as the within-subjects factor and IAT scores as the dependent variable. To assess the moderating roles of mental toughness and biological sex, mixed-design ANCOVAs were performed, with sex as a between-subjects factor and MT as a continuous covariate. To ensure the interpretability of the main effects within the ANCOVA models, the MT covariate was mean-centered prior to the analyses. Prior to hypothesis testing, the assumptions for repeated-measures analyses were rigorously evaluated; specifically, the assumption of sphericity was assessed using Mauchly’s test, and the Greenhouse–Geisser correction was applied to the degrees of freedom whenever this assumption was violated. The assumption of homogeneity of regression slopes was formally tested by examining the interaction terms between the factors and the continuous covariate. All respective interaction effects were non-significant, confirming that the regression slopes were homogeneous across groups and protocols, thereby validating the use of the ANCOVA models. Furthermore, to control for the family-wise error rate associated with running multiple ANOVA and ANCOVA models on the same dependent variable, a Bonferroni adjustment was applied to the significance threshold, establishing a stricter significance threshold of *p* < 0.017.

Partial eta-squared (η^2^_p_) calculations were used to determine the effect size of each running percentage of maximal speed in both conditions with or without MT as a covariate. For estimating the effect magnitude η^2^_p_ was interpreted using conventional thresholds in which values from 0.01 to less than 0.06 indicate a small effect, values from 0.06 to less than 0.14 indicate a medium effect, and values of 0.14 and higher indicate a large effect [29].

## 3. Results

Descriptive statistics for the female and male subsamples are presented in Table 1. The nonparametric Shapiro–Wilk test showed no significant deviations from normality.

Males significantly (*p* < 0.001) outperformed females in all IAT testing conditions, while there were no significant differences in MT or in the relative effects of running, except for aerobic running at 95% of maximum.

Results of analyses of variance (RM ANOVA, RM ANCOVA, and Mixed ANCOVA) with anaerobic running at different intensities (SR300y95, SR300y90, SR300y85, and SR300y80) as a repeated-measures factor, MT as a covariate, and sex as a between-subjects factor are presented in Table 2.

Results of the RM ANOVA revealed a significant large effect of anaerobic running speed on IAT performance (η^2^_p_ > 0.14). After including mental toughness as a covariate, the main effect of running speed remained statistically significant and maintained a large magnitude (η^2^_p_ > 0.14). In contrast, sex demonstrated a significant, large between-subjects effect on IAT performance (η^2^_p_ > 0.14), whereas the between-subjects effect of mental toughness, as well as all interaction effects, remained non-significant.

Results of analyses of variance (RM ANOVA, RM ANCOVA, and Mixed ANCOVA) with aerobic running in different intensities (CT2.4kmmax, CT2.4km95) as a repeated-measure factor, MT as a covariate and sex as a between-subjects factor are presented in Table 3.

Results of the RM ANOVA revealed a significant large effect of aerobic running speed on IAT performance η^2^_p_ > 0.14). After the inclusion of mental toughness as a covariate, the main effect of running speed remained statistically significant and maintained a large magnitude (η^2^_p_ > 0.14). In contrast, sex demonstrated a significant large between-subjects effect on IAT performance η^2^_p_ > 0.14), whereas the between-subjects effect of mental toughness, as well as all interaction effects, remained non-significant.

## 4. Discussion

The primary aim of this study was to examine the impact of physical exertion at varying intensities (submaximal and maximal anaerobic and aerobic running) on subsequent change-of-direction speed (CODS) performance among police students, while exploring the role of mental toughness (MT) and biological sex. The findings support the initial hypothesis regarding the fatiguing protocols, showing that progressive physical exertion impairs subsequent CODS performance. The main effects of running speed remained statistically significant after adjusting for mental toughness in the repeated-measures ANCOVA and mixed repeated-measures ANCOVA models. The results indicate that mental toughness has little to no effect on the physiological rate of decline. Additionally, biological sex was identified as a significant between-subjects factor: male police students outperformed female students across protocols, while both sexes showed a proportional fatigue-induced performance decline. However, it should be noted that the majority of the results obtained do not support the second and third hypotheses. Analyzing the full context of the research can still provide a basis for further research on this topic.

The descriptive characteristics of the sample (Table 1) highlight the participants’ high levels of physical and psychological preparedness. The police students demonstrated substantial physical fitness, an expected outcome given their successful completion of rigorous recruitment protocols and regular attendance at physical education training. Furthermore, the participants demonstrated exceptionally high MT levels, with average scores consistently approaching the upper limit of the scale for both sexes. A notable observation in the data is the low standard deviation of these scores, indicating a pronounced homogeneity in the sample’s psychological resilience. This uniformity strongly aligns with findings from recent investigations involving similar tactical populations, which suggest that individuals who successfully navigate stringent law enforcement selection processes share inherent psychological traits. Specifically, candidates with lower psychological hardiness and emotional stability are typically filtered out early in the recruitment phase, resulting in a highly uniform cohort [30]. Consequently, the homogenized MT observed in this sample accurately reflects the specialized profile of police students who are selected precisely for their capacity to withstand both physical and cognitive stressors during high-intensity tasks.

Comparison by biological sex (Table 1) revealed a significant effect on absolute IAT, with male students consistently achieving faster completion times than females. This variation aligns with established physiological and tactical literature detailing sexual dimorphism in body composition, anthropometric characteristics, and absolute power output, which directly facilitates more efficient acceleration and deceleration mechanics during high-intensity movements [3,5]. However, a more nuanced perspective emerges when examining the relative effects of fatigue on CODS (i.e., the percentage of performance decrement). Despite clear discrepancies in baseline absolute speed, the results showed no statistically significant differences between the sexes in the relative decline in performance across almost all anaerobic and aerobic running conditions. This phenomenon directly replicates the findings of previous studies [4], confirming that when physical degradation is normalized against baseline capacities, the relative trajectory of performance deterioration remains parallel, indicating that central and peripheral metabolic fatigue pathways operate uniformly across biological sexes. The only exception occurred after submaximal aerobic running at 95% intensity, where males exhibited a significantly greater relative decline than females. No significant differences were observed regarding MT. This lack of psychological divergence can be attributed to stringent law-enforcement recruitment criteria and rigorous institutional selection processes, which inherently favor high emotional stability, hardiness, and resilience regardless of biological sex [7,31,32]. Taking all findings from Table 1 into account, it can be concluded that while biological sex dictates the baseline capacities for absolute speed and agility due to physiological advantages, it generally does not dictate the relative rate at which these capacities deteriorate under acute exhaustion, nor does it influence underlying psychological resilience. Consequently, male and female police students exhibit a highly uniform psychological threshold and parallel psychophysiological responses under severe homeostatic stress.

The results obtained under both anaerobic and aerobic running conditions revealed a highly consistent pattern (Table 2 and Table 3). In line with the first hypothesis and previous findings in police [4], increasing exercise intensity was associated with poorer subsequent CODS performance, indicating that both anaerobic and aerobic fatigue negatively affected IAT performance. Similar findings have been reported in athletic and tactical populations, where accumulated metabolic stress, impaired neuromuscular function, and reduced force-production capacity contribute to decrements in change-of-direction performance following strenuous exercise [2,33,34,35,36]. Therefore, the present findings further support the notion that fatigue-induced reductions in CODS represent a robust and reproducible phenomenon across different exercise modalities.

Contrary to the second and third hypotheses, neither the interaction between MT and exercise intensity nor the interaction between sex and exercise intensity was statistically significant in the repeated-measures models under either anaerobic or aerobic conditions. These findings suggest that neither MT nor sex altered the fundamental rate of CODS deterioration with increasing exercise intensity. Furthermore, MT did not emerge as a significant independent between-subject predictor in these models. Such findings are somewhat inconsistent with studies reporting positive associations between MT and performance maintenance under demanding physical conditions [6,10,11]. However, the existing literature has primarily focused on endurance performance, persistence, pain tolerance, or perceived exertion rather than on agility-related performance following acute metabolic fatigue. Consequently, the mechanisms by which MT may influence performance could vary depending on the specific physical task and energy systems examined.

Although no statistically significant main or interaction effects involving MT were observed in the univariate repeated-measures models, several aspects of the findings warrant consideration. First, the introduction of MT as a covariate clearly demonstrated that the main effect of exercise intensity remained highly significant. This confirms that while MT may be associated with overall functional capacity, it does not override or neutralize the profound physiological reality of acute neuromuscular fatigue. Second, the descriptive statistics revealed exceptionally high and relatively homogeneous MT scores across both sexes. Similar homogeneity has previously been reported among police recruits [30]. Such restricted variability may have substantially reduced the statistical power to detect meaningful individual differences and interaction effects.

An additional explanation may be derived from contemporary psychobiological perspectives on exercise performance. According to the psychobiological model [13], psychological factors influence performance primarily through their effects on motivation, perceived effort, and the decision to continue exerting effort, rather than through direct modulation of peripheral physiological fatigue mechanisms. Supporting this view, experimental evidence has demonstrated that mental fatigue can impair physical performance by increasing perceived exertion even in the absence of substantial physiological changes [14]. Within this framework, MT may be expected to exert a greater influence on endurance performance, exercise tolerance, or persistence during prolonged effort than on preserving agility-based performance once considerable neuromuscular fatigue has already developed. Given that the IAT relies heavily on rapid force production, acceleration, deceleration, and change-of-direction mechanics, the performance decrements observed in the present study may have been predominantly constrained by physiological rather than psychological factors. This interpretation may help explain why MT did not significantly moderate the rate of CODS decline, despite its documented associations with performance resilience in other physical performance settings.

Taken together, the present findings provide mixed support for the initial hypotheses. Namely, the observed deterioration in CODS performance following progressively more demanding anaerobic and aerobic running strongly supports the first hypothesis. Conversely, the empirical data preclude the confirmation of the second and third hypotheses. The parallel rate of performance decline observed between male and female students, alongside the non-significant role of MT, demonstrates that while baseline absolute capacities differ by sex, the relative psychophysiological trajectory of acute physical degradation operates uniformly across this tactical population.

Several limitations should be acknowledged when interpreting the findings of this study. First, the exceptionally high homogeneity of the specialized sample, consisting exclusively of police students with highly uniform baseline physical readiness and MT scores, limited the overall data variance and the statistical power needed to detect subtle interaction effects across the different fatigue gradients. Second, although justified by power analysis, this sample remains too small, especially in terms of the number of female subjects. Furthermore, the sample may not be fully representative of the broader law enforcement population, limiting the generalizability of the present findings to older officers, other ranks, or other specialized tactical units. Moreover, the IAT, although highly reliable and contextually valid for tactical occupations, is a pre-planned CODS test that does not capture the perceptual-cognitive and reactive decision-making demands inherent to real-world law enforcement operations. Future research should expand on these findings by evaluating more diverse tactical populations, including active-duty police officers of varying ages and ranks, as well as specialized operational units, to enhance the model’s external validity. Longitudinal studies could track whether structured mental resilience protocols or targeted psychological skills training actively alter the rate of performance decline under severe physical strain over time. Another limitation was asymmetry in the testing protocols, driven by logistical reasons stands as a limitation of the present study that should be addressed in future research. Furthermore, when interpreting the overall performance trends across both running modalities, a direct comparison between the absolute maximal aerobic and maximal anaerobic fatigue states on CODS could not be established. Because the agility test was safely omitted following the maximal anaerobic protocol, any comparative conclusions regarding the relative impact of these two metabolic domains must be restricted to submaximal intensities or treated as general directional trends. While both pathways clearly demonstrate a robust, intensity-dependent degradation of agility, the lack of post-maximal anaerobic data prevents a definitive determination of which energy system fatigue exerts a more severe absolute impairment on technical agility performance. In the end, because the participants exhibited exceptionally high and homogeneous MT scores, the study effectively examined a narrow range of psychological characteristics. Consequently, the findings cannot be generalized to broader police populations or other tactical personnel with greater variability in psychological profiles. That is why future studies should examine more diverse samples.

The practical implications of this study are relevant for the design of tactical conditioning and operational training programs. The findings demonstrate that both anaerobic and aerobic fatigue substantially impair subsequent change-of-direction speed performance, highlighting the importance of incorporating fatigue-specific training stimuli into police education and occupational preparation. Training scenarios should therefore include high-intensity running tasks followed immediately by agility-based and occupation-specific activities to better reflect the physical demands encountered during law enforcement operations. Furthermore, although male participants consistently achieved superior absolute CODS performance, the relative rate of fatigue-induced performance decline was largely comparable between sexes. This finding suggests that fatigue-management strategies and conditioning approaches aimed at maintaining performance under physical strain may be applied similarly across male and female police students. Given the uniformly high levels of mental toughness observed within this sample and the absence of significant effects in the repeated-measures models, the present findings do not support the notion that mental toughness independently mitigates the physiological decline in CODS performance under acute fatigue.

## Figures and Tables

**Table 1:** Descriptive characteristics of the study participants and differences between sexes.

	Female (n = 11)				Male (n = 19)			
	Mean	Standard Deviation	Minimum	Maximum	Mean	Standard Deviation	Minimum	Maximum
IATinitial ***	19.80	0.93	17.90	21.30	17.30	0.93	15.90	19.40
IATPost300y80 ***	20.10	1.10	17.70	21.50	17.50	0.81	16.40	19.20
IATPost300y85 ***	20.80	1.11	19.00	22.40	18.20	0.65	17.00	20.00
IATPost300y90 ***	21.20	1.26	18.60	23.00	18.90	0.87	17.60	20.60
IATPost300y95 ***	21.10	1.12	19.00	22.80	18.80	0.89	17.70	21.30
IATPostCT95 ***	21.00	1.53	18.80	22.90	18.90	1.05	17.50	21.30
IATPostCTmax ***	21.30	1.34	19.20	24.00	18.70	1.18	17.30	21.90
EfIATPost300y95	6.48	2.77	1.54	10.15	8.94	3.88	0.43	14.97
EfIATPost300y90	6.98	3.81	0.91	12.47	9.37	3.98	2.68	17.74
EfIATPost300y85	5.12	4.66	−0.75	13.05	5.78	3.20	0.59	12.23
EfIATPost300y80	1.31	5.32	−6.33	13.29	1.75	2.96	−2.53	8.84
EfIATPostCTmax	7.37	5.83	0.99	21.85	8.68	5.44	−2.20	18.28
EfIATPostCT95 *	5.79	5.10	−2.34	12.21	9.43	3.84	−0.29	17.76
Mental Toughness	6.30	0.44	5.63	6.88	6.42	0.52	5.38	7.00

Note: * *p* < 0.05, *** *p* < 0.001.

**Table 2:** Effects of anaerobic running, mental toughness, and sex on Illinois Agility Test performance.

**Within Subjects**	**Sum of Squares**	**df**	**Mean Square**	**F**	**p**	**η^2^_p_**
Running Speed	56.00	4.00	14.00	56.70	<0.001	0.661
Residual	28.70	116.00	0.25			
Running Speed	55.97	4.00	13.99	57.52	<0.001	0.673
Running Speed × MT	1.41	4.00	0.35	1.45	0.22	0.049
Residual	27.25	112.00	0.24			
Running Speed	49.96	4.00	12.49	50.61	<0.001	0.652
Running Speed × Sex	1.58	4.00	0.40	1.60	0.18	0.056
Running Speed × MT	0.59	4.00	0.15	0.60	0.66	0.022
Residual	26.65	108.00	0.25			
**Between Subjects**	**Sum of Squares**	**df**	**Mean Square**	**F**	**p**	**η^2^_p_**
Residual	311.00	29.00	10.70			
MT	2.29	1.00	2.29	0.21	0.65	0.007
Residual	308.39	28.00	11.01			
Sex	212.43	1.00	212.43	59.78	<0.001	0.69
MT	0.11	1.00	0.11	0.03	0.86	0.001
Residual	95.95	27.00	3.55			

Note: Running Speed-RM Factor, MT—mental toughness, df—degrees of freedom, F—statistic, p—statistical significance, η^2^_p_—effect size.

**Table 3:** Effects of aerobic running, mental toughness, and sex on Illinois Agility Test performance.

**Within Subjects**	**Sum of Squares**	**df**	**Mean Square**	**F**	**p**	**η^2^_p_**
Running Speed	42.60	2.00	21.32	47.50	<0.001	0.621
Residual	26.10	58.00	0.45			
Running Speed	42.66	2.00	21.33	46.01	<0.001	0.622
Running Speed × MT	0.09	2.00	0.05	0.10	0.90	0.004
Residual	25.96	56.00	0.46			
Running Speed	37.93	2.00	18.97	40.82	<0.001	0.602
Running Speed × Sex	0.04	2.00	0.02	0.04	0.96	0.002
Running Speed × MT	0.87	2.00	0.44	0.94	0.40	0.034
Residual	25.09	54	0.46			
**Between Subjects**	**Sum of Squares**	**df**	**Mean Square**	**F**	**p**	**η^2^_p_**
Residual	207.00	29.00	7.13			
MT	1.34	1.00	1.34	0.18	0.67	0.006
Residual	205.48	28.00	7.34			
Sex	120.19	1.00	120.19	38.05	<0.001	0.585
MT	0.05	1.00	0.05	0.02	0.90	0.001
Residual	85.29	27.00	3.16			

Note: Running Speed-RM Factor, MT—mental toughness, df—degrees of freedom, F—statistic, p—statistical significance, η^2^_p_—effect size.

## Data Availability

Data is available upon request from filip.kukic@gmail.com or milosmilosevic80@yahoo.com.

## References

[1] Sheppard J.M., Young W.B. (2006). Agility Literature Review: Classifications, Training and Testing. J. Sports Sci..

[2] Joseph A., Wiley A., Orr R., Schram B., Dawes J.J. (2018). The Impact of Load Carriage on Measures of Power and Agility in Tactical Occupations: A Critical Review. Int. J. Environ. Res. Public Health.

[3] Kukić F., Koropanovski N., Janković R., Čvorović A., Dawes J.J., Lockie R.G., Orr R.M., Dopsaj M. (2020). Association of Sex-Related Differences in Body Composition to Change of Direction Speed in Police Officers While Carrying Load. Int. J. Morphol..

[4] Koropanovski N., Orr R.M., Dopsaj M., Heinrich K.M., Dawes J.J., Kukić F. (2022). Effects of Maximal and Submaximal Anaerobic and Aerobic Running on Subsequent Change-of-Direction Speed Performance among Police Students. Biology.

[5] Orr R.M., Kukić F., Čvorović A., Koropanovski N., Janković R., Dawes J.J., Lockie R.G. (2019). Associations between Fitness Measures and Change of Direction Speeds with and without Occupational Loads in Female Police Officers. Int. J. Environ. Res. Public Health.

[6] Gameiro N., Rodrigues F., Antunes R., Matos R., Amaro N., Jacinto M., Monteiro D. (2023). Mental Toughness and Resilience in Trail Runner’s Performance. Percept. Mot. Ski..

[7] Gucciardi D.F., Hanton S., Gordon S., Mallett C.J., Temby P. (2015). The Conceptualization of Mental Toughness Tests of Dimesionality, Nomological Network, and Traitness. J. Pers..

[8] Clough P., Strycharczyk D. (2012). Developing Mental Toughness: Improving Performance, Wellbeing and Positive Behaviour in Others.

[9] Gucciardi D.F., Stamatis A., Ntoumanis N. (2017). Controlling Coaching and Athlete Thriving in Elite Adolescent Netballers: The Buffering Effect of Athletes’ Mental Toughness. J. Sci. Med. Sport.

[10] Crust L., Clough P.J. (2005). Relationship between Mental Toughness and Physical Endurance. Percept. Mot. Ski..

[11] Nicholls A.R., Polman R.C.J., Levy A.R., Backhouse S.H. (2008). Mental Toughness, Optimism, Pessimism, and Coping among Athletes. Personal. Individ. Differ..

[12] Knicker A.J., Renshaw I., Oldham A.R.H., Cairns S.P. (2011). Interactive Processes Link the Multiple Symptoms of Fatigue in Sport Competition. Sports Med..

[13] Marcora S.M. (2008). Do We Really Need a Central Governor to Explain Brain Regulation of Exercise Performance?. Eur. J. Appl. Physiol..

[14] Marcora S.M., Staiano W., Manning V. (2009). Mental Fatigue Impairs Physical Performance in Humans. J. Appl. Physiol..

[15] Hsieh Y.-C., Lu F.J.H., Gill D.L., Hsu Y.-W., Wong T.-L., Kuan G. (2024). Effects of Mental Toughness on Athletic Performance: A Systematic Review and Meta-Analysis. Int. J. Sport Exerc. Psychol..

[16] Gába A., Kapuš O., Cuberek R., Botek M. (2015). Comparison of Multi- and Single-Frequency Bioelectrical Impedance Analysis with Dual-Energy X-Ray Absorptiometry for Assessment of Body Composition in Post-Menopausal Women: Effects of Body Mass Index and Accelerometer-Determined Physical Activity. J. Hum. Nutr. Diet..

[17] Barringer N.D., McKinnon C.J., O’Brien N.C., Kardouni J.R. (2019). Relationship of Strength and Conditioning Metrics to Success on the Army Ranger Physical Assessment Test. J. Strength Cond. Res..

[18] Streetman A., Paspalj D., Zlojutro N., Bozic D., Dawes J.J., Kukic F. (2022). Association of Shorter and Longer Distance Sprint Running to Change of Direction Speed in Police Students. NBP Nauka Bezb. Polic..

[19] White M., Sevene T., Adams K. (2015). The Reliability of the 300-Yard Shuttle Run in High School Girls Basketball Players. J. Sports Sci..

[20] Sales M.M., Sousa C.V., da Silva Aguiar S., Knechtle B., Nikolaidis P.T., Alves P.M., Simmes H.G. (2019). An Integrative Perspective of the Anaerobic Threshold. Physiol. Behav..

[21] Goodwin M.L., Harris J.E., Hernandez A., Gladden L.B. (2007). Blood Lactate Measurements and Analysis During Exercise: A Guide for Clinicians. J. Diabetes Sci. Technol..

[22] Lockie R.G., Hernandez J.A., Moreno M.R., Dulla J.M., Dawes J.J., Orr R.M. (2020). 2.4-Km Run and 20-m Multistage Fitness Test Relationships in Law Enforcement Recruits After Academy Training. J. Strength Cond. Res..

[23] Alvero-Cruz J.R., Girldez Garcia M.A., Carnero E.A. (2017). Reliability and Accuracy of Coopers Test in Male Long Distance Runners. Rev. Andal. Med. Deporte.

[24] Hachana Y., Chaabone H., Nabli M.A., Attia A., Moualhi J., Farhat N., Elloumi M. (2013). Test-Retest Reliability, Criterion-Related Validity, and Minimal Detectable Change of the Illinois Agility Test in Male Team Sport Athletes. J. Strength Cond. Res..

[25] Stamatis A., Deal P.J., Morgan G.B., Forsse J.S., Papadakis Z., McKinley-Barnard S., Scudamore E.M., Koutakis P. (2020). Can Athletes Be Tough yet Compassionate to Themselves? Practical Implications for NCAA Mental Health Best Practice No. 4. PLoS ONE.

[26] Stamatis A., Morgan G.B., Spinou A., Tsigaridis K.G. (2023). Mental Toughness and Osteoarthritis: Postsurgery Improvement in Knee Pain/Functionality in Older Adults. Rehabil. Psychol..

[27] Milošević M., Božović B., Dopsaj M. (2022). Psychometric Properties of the Serbian Version of Mental Toughness Inventory and Dark Triad Dirty Dozen in Police Students. Nauka Bezb. Polic..

[28] Hawk S.T., Keijsers L., Branje S.J.T., Graaff J.V.D., Wied M.D., Meeus W. (2013). Examining the Interpersonal Reactivity Index (IRI) Among Early and Late Adolescents and Their Mothers. J. Personal. Assess..

[29] Richardson J.T.E. (2011). Eta Squared and Partial Eta Squared as Measures of Effect Size in Educational Research. Educ. Res. Rev..

[30] Risan P., Skoglund T.H., Sandvik A.M., Milne R. (2022). Personality and Hardiness among Police Students: An Evaluative Pilot Study. Nord. J. Stud. Polic..

[31] Cowden R.G., Meyer-Weitz A. (2016). Mental Toughness in South African Competitive Tennis: Biographical and Sport Participation Differences. Int. J. Sport Exerc. Psychol..

[32] Papageorgiou K.A., Benini E., Bilello D., Gianniou F.M., Clough P.J., Costantini G. (2019). Bridging the Gap: A Network Approach to Dark Triad, Mental Toughness, the Big Five, and Perceived Stress. J. Personal..

[33] Sporis G., Vucetic V., Milanovic L., Milanovic Z., Krespi M., Krakan I. (2014). A Comparison of Anaerobic Endurance Capacity in Elite Soccer, Handball and Basketball Players. Kinesiology.

[34] Hunter S.K. (2016). Sex Differences in Fatigability of Dynamic Contractions. Exp. Physiol..

[35] Arthur C.A., Fitzwater J., Hardy L., Beattie S., Bell J. (2015). Development and Validation of a Military Training Mental Toughness Inventory. Mil. Psychol..

[36] Pageaux B., Lepers R. (2018). The Effects of Mental Fatigue on Sport-Related Performance. Progress in Brain Research.

